# Implementing guidelines and training initiatives to improve cross-cultural communication in primary care consultations: a qualitative participatory European study

**DOI:** 10.1186/s12939-017-0525-y

**Published:** 2017-02-10

**Authors:** E. Teunissen, K. Gravenhorst, C. Dowrick, E. Van Weel-Baumgarten, F. Van den Driessen Mareeuw, T. de Brún, N. Burns, C. Lionis, F. S. Mair, C. O’Donnell, M. O’Reilly-de Brún, M. Papadakaki, A. Saridaki, W. Spiegel, C. Van Weel, M. Van den Muijsenbergh, A. MacFarlane

**Affiliations:** 1 Department of Primary and Community Care, Radboud University Medical Center, NIjmegen, the Netherlands; 2 Department of Psychological Sciences, B121 Waterhouse Buildings University of Liverpool, Liverpool, United Kingdom; 3 Discipline of General Practice, School of Medicine, National University ofIreland, Galway, Ireland; 4 Faculty of Health and Medicine, Lancaster University, Lancaster, UK and General Practice & Primary Care, Institute of Health & Wellbeing, College of MVLS, University of Glasgow, Glasgow, UK; 5 Clinic of Social and Family Medicine, University of Crete Medical School, Crete, Greece; 6 General Practice & Primary Care, Institute of Health and Wellbeing, College of Medical Veterinary and Life Sciences, University of Glasgow, Glasgow, UK; 7 Clinic of Social and Family Medicine, University of Crete Medical School, Crete, Greece; 8 Department of Social Work, School of Health and Social Welfare Technological Educational Institute of Crete Heraklion, Crete, Greece; 9 Centre for Public Health, Medical University of Vienna, Kinderspitalgasse 15/1st floor, A-1090 Vienna, Austria; 10 Department of Primary and Community Care, Radboud University Medical Center, Nijmegen, the Netherlands; 11 Australian Primary Health Care Research Institute, Nijmegen, the Netherlands; 12 Department of Primary and Community Care, Radboud University Medical Center, Nijmegen, the Netherlands; 13 Graduate Entry Medical School, University of Limerick, Limerick, Ireland; 14 Pharos, Centre of Expertise for Health Disparities, Utrecht, the Netherlands

**Keywords:** Primary Health Care, Transients and Migrants, General Practice, Community-Based Participatory Research, Cross-cultural communication, Equity

## Abstract

**Background:**

Cross-cultural communication in primary care is often difficult, leading to unsatisfactory, substandard care. Supportive evidence-based guidelines and training initiatives (G/TIs) exist to enhance cross cultural communication but their use in practice is sporadic. The objective of this paper is to elucidate how migrants and other stakeholders can adapt, introduce and evaluate such G/TIs in daily clinical practice.

**Methods:**

We undertook linked qualitative case studies to implement G/TIs focused on enhancing cross cultural communication in primary care, in five European countries. We combined Normalisation Process Theory (NPT) as an analytical framework, with Participatory Learning and Action (PLA) as the research method to engage migrants, primary healthcare providers and other stakeholders. Across all five sites, 66 stakeholders participated in 62 PLA-style focus groups over a 19 month period, and took part in activities to adapt, introduce, and evaluate the G/TIs. Data, including transcripts of group meetings and researchers’ fieldwork reports, were coded and thematically analysed by each team using NPT.

**Results:**

In all settings, engaging migrants and other stakeholders was challenging but feasible. Stakeholders made significant adaptations to the G/TIs to fit their local context, for example, changing the focus of a G/TI from palliative care to mental health; or altering the target audience from General Practitioners (GPs) to the wider multidisciplinary team. They also progressed plans to deliver them in routine practice, for example liaising with GP practices regarding timing and location of training sessions and to evaluate their impact. All stakeholders reported benefits of the implemented G/TIs in daily practice. Training primary care teams (clinicians and administrators) resulted in a more tolerant attitude and more effective communication, with better focus on migrants’ needs. Implementation of interpreter services was difficult mainly because of financial and other resource constraints. However, when used, migrants were more likely to trust the GP’s diagnoses and GPs reported a clearer understanding of migrants’ symptoms.

**Conclusions:**

Migrants, primary care providers and other key stakeholders can work effectively together to adapt and implement G/TIs to improve communication in cross-cultural consultations, and enhance understanding and trust between GPs and migrant patients.

## Background

Effective communication is at the core of person-centred care. In situations where patients and doctors do not share language or culture even basic communication can become problematic, with detrimental effects on access, outcomes and safety [1–3]. With migration on the rise globally, problems with cross-cultural communication are increasingly common in primary care, leaving migrants vulnerable to sub-standard care [4–6] and healthcare providers unsatisfied with the quality of care they deliver [7–9]. Everyone has a fundamental right to health and to access health care, legally enshrined in both international and European instruments, such as the European Charter of Fundamental Rights [10]. Therefore, this form of health inequity urgently requires attention.

Primary care interventions can improve healthcare access, quality, health outcome and equity in health [11]. There are also guidelines and training initiatives (G/TIs) available that are designed to support communication in primary care cross cultural consultations, for example as developed in the EU funded MEMP-TI project (http://www.mem-tp.org/.] However, like many other clinical guidelines [12, 13] their implementation in routine practice remains patchy [14, 15]. One of the reasons being the fact that these G/TIs are often not well tailored to daily practice, and stakeholders (patient service users as well as primary care providers are often not involved in their development. Consequently, there is a reliance on informal strategies rather than the use of professional trained interpreters by appropriately trained healthcare providers: this knowledge-practice gap is under researched [15–18]. Raising the voices of marginalised communities is recognised as a key lever for effective intervention design, implementation and evaluation in primary care [19, 20]. Many national health policies are explicit about the value of community participation for practice improvement in primary care [21–23]. However, the involvement of migrants is rare as they are considered ‘hard-to-reach’ on the basis of inaccessibility, language discordance and cultural difference [24] and there are specific challenges involving undocumented migrants in health research [25]. Recent research provides evidence of the value of participatory methodologies to engage migrants and indicates the positive impact of their involvement on the development of guidelines to improve communication in cross-cultural consultations [14, 24]. Taken together, this shows that it is imperative to involve migrants in the *implementation* and *evaluation* of available G/TIs in primary care to improve communication in cross-cultural consultations. The need to address this has become a pressing issue following the vulnerabilities of the recent large influx into Europe of those who have been forcibly displaced [26].

The objective of this paper is to elucidate how migrants and other stakeholders can adapt, introduce and evaluate their selected G/TIs in daily clinical practice.

## Methods

### Study Design and setting

The RESTORE project was a qualitative, comparative case-study [27] that prospectively investigated and supported the implementation of G/TIs to improve communication for migrants in five primary care settings (Austria, England, Greece, Ireland and the Netherlands), using a novel combination of Normalisation Process Theory (NPT) and Participatory Learning and Action (PLA) methodology [16]. Policy analysis work was undertaken in a sixth country – Scotland.

A detailed description of the study protocol is available [28].

NPT is a theoretical framework concerned with the work that individuals and organisations have to carry out in order to embed and normalise new, complex ways of working into routine practice [29]. It has been widely used to guide the implementation of a variety of system improvements in primary care practice, [29] and alerts researchers and implementers to the realities of implementation in real time and the interactions that do, or do not, occur between the individuals and groups charged with that implementation, by focussing attention on four principal constructs (Table 1).

**Table 1:** NPT constructs

Construct	What it addresses
Sense-making	Can those involved in the implementation make sense of it?
Cognitive Participation	Do relevant stakeholders ‘buy into’ the implementation work? Can those involved maintain their involvement and get others involved and engaged?
Enacting	What has to be done to make the intervention being implemented work in routine practice?
Appraisal work	How can the intervention be monitored and evaluated? Can it be re-designed to sustain its use?

PLA creates a participatory space where stakeholders can work together democratically [25] on an implementation project. Our rationale for combining NPT and PLA is available elsewhere [30].

A comprehensive mapping process [14] in the six RESTORE countries identified twenty G/TIs that were designed to support communication in cross-cultural consultations. Research teams in five countries (Austria, England, Ireland, the Netherlands, and Greece) used NPT to appraise these G/TIs to create a smaller set of four or five G/TIs, selected for their likelihood of successful implementation in primary care in their countries [14]. Migrants and other key stakeholders in each setting were then invited to examine the set of G/TIs relevant to their setting and they successfully selected one as an implementation project for a primary care site in their region [31]. This paper focuses on the next stage of RESTORE: stakeholders’ participation in adaptation, delivery and evaluation of the selected G/TIs.

### Sampling and recruitment

Using purposive and network sampling [32], we recruited 66 stakeholders across five study sites to participate in adaptation, delivery and evaluation of the selected G/TIs, including migrant representatives, general practitioners (GPs), practice nurses, receptionists, practice assistants, practice managers, academics, interpreters, health service planners and policy makers (See Table 2). In each group diversity was reached regarding age and gender; migrants came from a large variety of different countries. Of these, 45 had been involved in the prior G/TI selection. All stakeholders had a good command of the national language at each fieldwork site.

**Table 2:** Participants in stakeholder groups

	Country				
	Austria^1^	England	Greece	Ireland	Netherlands
Migrants/migrant representatives	3	5	2	5	3
General practitioners	4	2	4	2	2
Primary care nurses	0	0	5	0	3
Primary care administrators	0	1	1	2	2
Interpreters/cultural mediators	0	0	0	3	1
Health service planners/policy makers/academics	1	2	7	1	1
Trainers	2	2	0	0	0
TOTAL	10	12	19	13	12

^1^In Austria, there were also three focus groups with migrants from Philippines, Turkey, and sub-Saharan Africa (*n =* 30) after the selection of a G TI was made

A commitment to engage multiple stakeholders in a meaningful participatory dialogue was at the core of the RESTORE project methodology. Overall this was successful as evidenced by the details of our sample. However there were challenges with various stakeholders in each setting which had to be addressed (see Table 3). Interestingly, dealing with these challenges was greatly helped by stakeholder engagement because stakeholders could draw on their knowledge of their own backgrounds (healthcare, community etc.) to help identify potential solutions to overcome challenges.

**Table 3:** Challenges in engaging stakeholders

Engaging migrant communities:
• Austria: GPs work in single handed practices without migrant patient representative groups. The research team fostered dialogue between academically-oriented stakeholders from primary care and migrant representatives.
• Greece & the Netherlands: GPs *engaged with migrants at project meetings in general but* expressed discomfort about involving migrant representatives directly in discussions about the practice. Research teams addressed this by engaging in parallel dialogues about these issues with migrant representatives and shared the information across the groups.
Engaging primary care staff:
• England: Restructuring of primary care made it difficult to involve a GP practice in the early stages of fieldwork. GP members of the stakeholder group offered their perspectives until a primary care team agreed to participate.
• Greece & the Netherlands: Healthcare staff found it difficult to attend long PLA focus groups. In the Netherlands research teams introduced shorter sessions; in Greece they met individually with practice staff.
• Ireland: Poor engagement of some GPs and administrators in the participating practice was offset by sustained commitment by the principal GP and practice manager.
Engaging interpreters:
• Greece: No formal primary care interpreting service existed. The research team made innovative arrangements with an NGO and a certified interpreter to negotiate telephone-based interpretation services for primary care patients.
• Ireland: There was no national interpreting service with trained interpreters. This was resolved by exploring and drawing on expertise of trained community interpreters within the stakeholder group.
Engaging policy makers and health service planners:
• England: Restructuring of primary care due to policy changes meant that a key policymaker stakeholder was moved to a different job. The stakeholder group brought in new policymakers/health service planners at a later stage of the project.

### Data generation and analysis

Data were generated and analysed throughout the process of implementation, which lasted between 15 and 19 months after stakeholders had selected a G/TI for their local setting. We used specific PLA techniques for data-generation in PLA informed focus groups. These focus groups consisted each time of the same stakeholders, as described in Table 2 (with minor variations depending on availability), to establish continuity in the adaptation, implementation and evaluation process. PLA techniques used included flexible brainstorming, direct ranking, card sort, seasonal calendar and speed evaluation (see Table 4). These have been used in primary care research previously, with the order they were used in designed to facilitate discussion and decision-making [24, 33].

**Table 4:** PLA techniques

Flexible Brainstorming	Fast and creative approach of using materials, such as pictures or objects, to generate information and ideas about the topic.
Direct Ranking	A transparent and democratic process that enables a group of stakeholders to indicate priorities or preferences.
Card sort	An interactive method for facilitating and recording brain storming around topics.
Seasonal calendar	Seasonal Calendar is a grid-based diagram used for co-operative planning and democratic decision-making. A flexible adaptive tool, it can be used as a ‘running record’ of stakeholder’s planning over time.
Speed evaluation	Speed evaluations are short verbal or written evaluations, often used at the end of a PLA session to indicate (to stakeholders and researchers alike) what key positive, negative and/or neutral experiences have occurred.

The data generated were then used to document details of stakeholders’ adaptation of their G/TIs, their planning to deliver them in daily practice and to evaluate their impact.

All researchers had prior experience of qualitative research and were trained in the use of PLA techniques [34]. A total of sixty two PLA style group meetings were held, with an average of twelve in each setting. Each meeting was audio-recorded and transcribed verbatim.

These data were subjected to a deductive qualitative analysis using an iteratively developed NPT coding frame (Table 5). At all sites at least 10% of all data were double coded with active attention for data that fell outside NPT. This paper focusses principally on two constructs of NPT: enacting (delivery in practice) and appraisal (evaluation) work and describes the results, challenges and solutions, according to the different stages of work: the engaging of stakeholders; the adaptation of the G/TIs to the local context; the delivering of the G/TIs in practice; and the evaluation of the implementation.

**Table 5:** NPT coding frame

NPT construct	Sense-making	Cognitive Participation	Enacting	Appraisal work
Descriptor	Stakeholders making sense of the G TIs presented to them Which ones do they think have value and benefit for their local setting? What are the positive and negative features of the G/TIs they examined? Were there shared views or not during this phase of thinking about the G/TIs?	Stakeholders’ engagement with the implementation project, process of direct ranking and selecting one G/TI to Process of selecting a G/TI reveals data about their motivation, willingness and perceived capacity to implement the G or TI, plus their feelings that it is right to be involved in the implementation work. Stakeholders thinking about other relevant stakeholders to enrol e.g., migrants, other practice staff members, trainers is part of the work of driving it forward Active enrolment work e.g., meeting someone to recruit them to the project	Stakeholders activities to introduce G Tis into clinical settings Planning imminent activities e.g. discussing divisions of labour about who makes a poster Information on what resources are available in their context to them to support these activities Stakeholders’ skills and participation in training to develop their skills Stakeholders fine tune their selected GTI to improve its quality or relevance for them Stakeholders implement knowledge from their selected G Tis into practice Divisions of labour to progress the implementation project e.g. who does what to enrol other stakeholders, to ensure smooth running of an interpreted consultation Descriptions of trying out new ways of working/communication with migrants and how it felt to apply the new way of working in e.g., consultations – did stakeholders have confidence in it? Data about the length of consultations and whether the new ways of working had positive or negative effects on consultations in practice	Stakeholders’ appraising the impact of the new way of working preferably after a period of use Stakeholders’ discussions about planning formal appraisals e.g. setting up exit interviews or patient questionnaires to monitor impact; collecting data on number of interpreted consultations Stakeholders’ reflections on findings from these appraisals, informal appraisals also relevant Can the new way of working be sustained in practice and what changes do stakeholders think are necessary to the service to ensure this?

In addition to the transcript data, each research team completed 5 fieldwork reports with standardised headings in English, that were created to monitor fieldwork and to facilitate the comparative analysis. These reports contained rich narrative descriptions of each of the implementation processes. The content of the reports was derived from a variety of sources, including PLA style focus groups, transcripts and data displays, team meeting minutes and fieldwork debriefings. The monitoring reports were discussed at RESTORE team meetings, encouraging a process of iterative reflection and cross-country comparison and exchange. Issues around the coding procedure and coding frame were discussed across the RESTORE teams, both face to face and by teleconference. These combined activities were important for contextualising and synthesising knowledge of the transcript data from across settings.

## Results

### Adapting G/TI to local context

The process of adaptation was more substantial and far-reaching than we anticipated, especially in Austria (TI) and England (TI). Table 6 charts the changes that stakeholders made to the selected G/TIs in each setting.

**Table 6:** Adapting G/TIs for local settings – Key changes

Original G/TI	Adapted G/TI
Austria	
Training Initiative: New European migrants and the NHS: Learning from each other, Manual for Trainers, First Edition February 2009, NHS Lothian, Dermot Gorman, Scotland	
• Aimed at community health professionals, GPs and clinical support staff • Content specific to Eastern European migrants in Scotland • Material on a broad range of healthcare issues including pregnancy and midwifery • E-learning module	• Aimed at GPs • Content adapted to Turkish, African and Arabic migrants in Austria • Material focused on healthcare issues relevant to GPs • Lectures, quality circles and e-learning module
England	
Training Initiative: Ears of Babel: Culturally sensitive primary healthcare, Pharos, Netherlands	
• One training session (4 hours) • Aimed at GPs only • Delivered by GP trainer & Migrant trainer • Focus on palliative care • Presentation, role play, group discussion	• Two training sessions (1½ hour, 2½ hours) • Aimed at multidisciplinary practice team • Delivered by professional drama based training company • Focus on mental health • Actor performed scenarios & adapted role play, group discussion
Greece	
Guideline: Guidance for communication in cross-cultural general practice consultations: Developed using a participatory research approach, Discipline of General Practice, Centre for Participatory Strategies, Health Services Executive & The Health Research Board, Ireland	
• Developed in setting with established face-to-face interpretation services	• Introduced in setting without face-to-face interpretation services • Setting up telephone interpretation service
Ireland	
Guideline and Training Initiative: Working with an interpreter is easy: Self-directed training package for health professionals, SPIRASI, Ireland	
• Aimed at health professionals only • Apparent acceptance of the use of informal interpreters in certain circumstances • Lack of detailed information about the dynamics of culture	• Aimed at inter-stakeholder multi-cultural multi-disciplinary group • Agreement on need to use formal interpreters • Additional training session on the dynamics of culture • Complemented by PLA style ‘Walk-Through’ to allow stakeholders to practise the application of knowledge from training into practice
The Netherlands	
Training Initiative: “Did I explain it clearly?” How to communicate with migrants with lower education and less command of the Dutch language, Pharos, The Netherlands	
• One training session (4 hours) • Aimed mainly at medical practice assistants • Use of formal interpreters • Focus on migrants with limited education and command of the Dutch language	• Two training sessions (4 hours, 3 hours) • Aimed at entire practice team (including GPs and practice nurses) • Use of formal and informal interpreters • Focus on migrants and natives with limited education and command of the Dutch language • Developing ‘improvement plans’ with GP practice • Regular evaluations of the impact of the training

The flexible and democratic approach of the PLA methodology allowed us to work with stakeholders to adapt the selected G/TIs to the local context. Stakeholders built shared knowledge and confidence in each other’s expertise which aided their decision-making about the adaptations.

Adaptation was not limited to the settings where a G/TI was selected from another country (as happened in Austria (TI), England (TI) and Greece (G)) but was also required in the settings where a national G/TI had been selected (Ireland (TI&G) and the Netherlands (TI)). The nature of the adaptations related to the target group, content, mode of delivery and trainers for the G/TIs.

#### Adapting target group

Across most settings stakeholders chose to expand the intervention from a focus on GPs alone to all general practice staff: the stakeholder group discussions made it clear that cross-cultural communication is a collective, and not an individual responsibility, which requires joint efforts for the practice to speak with the same voice. In Austria, where GPs work single-handed practices, this was impractical.

#### Adapting content

In England the stakeholder group chose to change emphasis of the TI from palliative care to mental health as they felt this would increase its relevance locally. In Ireland the original training on working with a professional interpreter had to be extended as a GP stakeholder did not feel fully equipped after the initial training to deliver interpreted consultations. Stakeholders worked together to design a role-played ‘walk-through’ consultation with a professional interpreter member of the stakeholder group. After consultation, the GP’s response was:

*“I’ve clearly never had it [the services of a professional interpreter], it’s brilliant! [….] The thoughts of doing that [consultation] with someone with broken English, with or without a friend, a non-professional interpreter, would be a nightmare. It would take so long to deal with one problem and so many people in the waiting room! (Ire, GP, SH1)*

Adaptation work of the training in the Netherlands was fundamentally driven by a decision of the Dutch government to withdraw funding for formal interpreters in primary care. As a result Dutch GPs wanted to know how they could work with informal interpreters and this was included in the training.

#### Adapting mode of delivery

In both the Netherlands and England stakeholder groups decided to split the 4 h training session of the original TI into two shorter sessions to leave scope for an iterative cycle of reflection, evaluation and improvement and also to fit in with GP practice preferences for shorter training sessions, due to time pressures in their surgeries.

#### Adapting trainers

Where the original TI was meant to be delivered by a GP and migrant trainer team, a migrant service representative (SH02) in the English site proposed inviting a local professional training company to deliver the training. After discussion the group agreed to involve the training company.

### Introducing G/TIs in practice

Overall, after the adaptation work, all four training courses were delivered in the RESTORE sites Austria, England, Ireland and the Netherlands. Following the guideline they had chosen to implement, new interpreting services were offered in two sites but recruitment challenges meant they were infrequently used, in Ireland, or not used at all in Greece (discussed below).

The introduction of the G/TIs required additional work:

Concerning the chosen Training Initiatives:

#### Logistics of delivering training

In Austria, England and the Netherlands stakeholders identified and liaised with suitable trainers developing training materials collaboratively and liaising extensively with GP practices regarding timing, duration and location of training sessions, necessary materials and refreshments.

Concerning the implementation of a guideline for interpretation services.

#### Resources for interpreted consultations

Both in Greece and in Ireland part of the implementation of the chosen guideline was the implementation of an interpretation service itself, as this was previously non-existent in the clinical settings involved. The stakeholders were involved in all aspects of setting up interpretation services from scratch, including identifying existing interpreting services, resources for using services and training the practice teams in using these interpreting services.

#### Advertising and recruiting for interpretation services

In Ireland the stakeholders realised there was limited ability to advertise services in the research site because, due to funding models for GP in Ireland other practices might perceive this as an attempt to ‘poach’ their patients which would reduce the income of other practices.

In Greece stakeholders discussed a potential problem with migrant worker’s employers attending consultations, and came to an agreed solution:

*Migrant: “I notice that many migrants come in with their boss, primarily the migrants that work in the fields and usually the boss tries to interpret. What will the GP do then? Will the service still be offered?” (Gr, MSU, SH14)*

*GP: “I think that we should ask the boss (…) to wait in the waiting area and we should use the phone line service only if okay with the patient. Does everyone agree with this? (..)(Gr, GP, SH6)*

*[All nod and say yes*]

### Evaluation

Stakeholders began planning the evaluation of their selected G/TI early in the implementation journey. They considered a wide range of potential strategies to formally evaluate the effectiveness of the intervention in practice and realised plans to gather data in four of the sites, using interviews, evaluation forms and e-mail communication with service users, healthcare providers and practice staff (see Table 7).

**Table 7:** Overview of Formal Evaluation of G/TIs on Practice

COUNTRY➔ Evaluation activity ↓ Number of:	Ireland Implementation of G &TI	England Implementation of TI	Austria Implementation of TI	The Netherlands Implementation of TI
Interviews to appraise impact on practice of the implemented G/TI	4 MSU 1 GP 1 I	1 PS		3 MSU
Evaluation Forms to appraise training and impact on practice	1 (M, GP, PS, I, H, T)		7 (GP)	15 (GP, PN, PS)
E-mails to appraise impact on practice				6 (GP, PN, PS)

*MSU = migrant service users*

*GP = General Practitioner*

*PN = primary care nurses, receptionists and practice assistants*

*PS = primary care administration/management staff*

*I = member of interpreting community*

*H = Health service planning and/or policy personnel*

After the G/TI had been implemented, stakeholders examined data from formal evaluations in PLA style focus groups devoted to evaluation and discussed their own perspectives on the worth of the G/TIs.

These sessions were highly valued by stakeholders, especially the contribution of the migrants: they flagged up issues the practice staff would not have considered, e.g. that the posters and leaflets in the practice were too difficult to read, and at the same time confirmed the positive effect of the selected G/TI.

Most stakeholders, in the evaluation stage including migrant service users as well as practice members who had not been members of the original stakeholder group, reported benefits of the implemented G/TI in daily practice. Focusing on the data from the formal evaluations, the training in cultural competencies and communication skills in the Netherlands and England led to positive effects in consultations, for example more effective communication in consultations between healthcare professionals and migrants with low literacy (GPs and practice nurses The Netherlands):

*“We are more aware of low literacy: we ask patients about it, take more time and arrange more support of social workers (..) we register it now in the patient record, and discuss consequences with the other practice members (The Netherlands, GP, SH4)*

Also in Austria participants experienced the training to be helpful.

*“I can transfer the lessons learned in the training into daily practice, especially in addressing mental health problems (Austria, GP, SH5)*

Training also led to a more tolerant and positive attitude towards migrant service users amongst receptionist staff:

*“I am now reacting much calmer than I did before, with more patience, when a migrant who doesn’t speak Dutch stands before me at the desk”. (The Netherlands, PN, SH5)*

*“The receptionists were all talking about the training which was good, because you know from that the other girls [receptionists] were saying positive things.” (England, PN, SH15)*

Even in Greece, despite the lack of uptake of the interpretation service, this positive shift towards migrant service users was noted:

*“RESTORE helped me open my eyes to my migrant patients and their needs, where in the past I just scanned over them.” (Greece,*/GP/SH6)

There were examples of increased flexibility in accommodating migrants’ appointments amongst all staff (England), and adaptation of the practice to the needs of low literate migrants (the Netherlands). Stakeholders in England and the Netherlands considered that these practice level changes were due to the fact that *all* clinical, managerial and reception practice members had been involved collectively in the training and thus shared the responsibility to implement the G/TI in the practice:

*“We could also go to a training low literacy together, and then go home, and then you have heard the information and that’s it. But we really have worked with each other, and therefore it is more relevant…” (The Netherlands, PN, SH5)*

The data about training in the use of interpreters in Ireland indicate improvements in consultations for the GP, interpreters and migrants involved.

The GP described advantages in terms of having a clearer picture of symptoms and more confidence in devising treatment plans:

*“So (..) I got a much truer picture of the type of symptoms she was having, and therefore [knew] which treatment to give her (….)[The last day] I gave her a treatment, without an interpreted consultation (..)(…).that wasn’t at all appropriate. So today we revised that, I told her to get rid of that prescription (…). (Ire, GP, SH1)*

The trained interpreter involved remarked on this positive feature of the consultation as well:

*“I think after the first few sentences she [the patient] actually realised that the communication is flowing and the fact that she’s speaking only Polish means she can focus on what she wanted to say. Not on how to translate it herself. And I think I could see that the difficulty she had was with medical terminology. There was a name of a medical condition that she had and that was interpreted - I think this is where I gained the trust.” (Ireland, interpreter, SH7)*

She also commented on the benefits of working with a GP and the fact that they had trained together.

Migrants’ comments on having better confidence in the GP’s diagnosis and treatment and a reported ease about having a ‘stranger’ i.e. the interpreter in the consultation.

*“In my case, it’s easy to trust when the interpreter is present, because I knew that she would be able to convey everything that I meant and that I would be understood. I did not feel any discomfort about it [the presence of the interpreter].” (Ireland MSU, SH2)*

Exploration of the reasons for lack of uptake of the newly established *interpreter service* as recommended by the Greek stakeholders’ selected guideline, provided valuable insights. Primary health care providers were keen to try this innovative new service and had overcome resource challenges to actually provide the service, but migrants identified barriers in implementation not previously foreseen: fear among migrants that it would cost them money or that they would have to use their own cellular phone for the telephone interpreter. Furthermore - despite the above mentioned intention of GPs to ask them to wait outside - migrants’ employers often attended their consultations to act as their interpreter. While migrants would have preferred a formal interpreter they felt obstructed to express this view.

Disadvantages of interpreted consultations mentioned in Ireland and Greece relate to practice level challenges – the lack of structural resources to provide interpreters, and logistic challenges organising triadic consultations and difficulties accessing trained interpreters.

The dominant challenges in each setting to sustaining the new ways of working recommended by the G/TIs in daily practice were time constraints that would undermine good intentions to continue new ways of working, or simply forgetting to keep a new practice going. While stakeholders could consider possible strategies for reconfiguration for these challenges, it was more difficult when the challenges related to lack of resources for on-going funding beyond the lifetime of RESTORE:

The lack of structural support, specifically finances for interpreter services, was a major barrier in Ireland and Greece. Another challenge identified during the evaluation in Greece was that migrants were unaware of their rights when it comes to their health and they themselves stated that ‘health care advocacy’ is essential in their community:

*“If the services available were clear to us migrants, on what rights we have and this was posted at the health centres, in many cases I wouldn’t bring someone with me. I am lost when I enter the health centre.” (Gr, MSU, SH3)*

Stakeholders were aware that these issues were related to local or national policy and anticipated that data from RESTORE could bring about changes to improve the organisational and contextual conditions to facilitate the sustained use of the G/TIs in practice.

## Discussion

### Summary of findings

The process of local adaptation of G/TIs was complex, time consuming but productive ensuring a tailored intervention for delivery and enhancing buy-in amongst stakeholders. Introduction of adapted G/TIs in practice settings involved intensive planning and problem solving about logistics, resources and advertising new services. Formal evaluations had to be carefully planned as well as ‘reflection space’ for stakeholders to collaboratively consider the impact of introducing G/TIs. Training resulted in a more tolerant attitude and more effective communication, with better focus on migrants’ needs. Implementation of interpreter services, when possible, led to more trust of migrants in doctors’ diagnoses and GPs reporting a clearer picture of migrants’ symptoms.

### Comparison with existing literature

This paper extends the existing literature [33, 35–37] by detailing the work required to implement G/TIs to improve communication between migrants and their primary care providers. It focuses attention on the need for *adaptation* of G/TIs to local context and on methods to achieve this. Our emphasis on the importance of adaptation sits well with the knowledge-to-action cycle proposed in the so-called ADAPTE initiative (www.adapt.org/). [38], although we consider that our commitment to comprehensive stakeholder involvement and our focus on collective problem-solving represent substantive advances over clinician-led adaptation processes. The involvement of migrants and other key stakeholders in evaluation is rare and our study demonstrates a positive impact in the field of migrant healthcare.

Like other interventions, the *introduction* of the selected G/TIs was challenging work that required attention to resources, skills and training and people’s confidence and trust in the new ways of working (29). Stakeholders overcame a range of issues to try out new knowledge in practice and to see if it would help them achieve their goals in primary care.

In relation to *evaluation*, our analysis resonates with the findings of earlier research that cultural competency training improves knowledge, attitudes and skills of GPs and practice nurses [39–41] and results in more patient satisfaction [40, 42, 43]. The involvement of all practice members, including reception staff and practice assistants, made it possible to clarify long-term agreements about improvements in their local settings for daily practice. These are novel findings in relation to implementation of G/TIs in this field and resonate with previous research that inter-professional collaborative agreements are needed to effectively change practice long-term [34, 44, 45].

By focusing on these different forms of implementation work, we have drawn attention to the ways in which some problems could be resolved by stakeholders themselves (e.g. enhancing their skill sets by designing additional training) while others could not (e.g. changes in national policy funding models for primary care and interpreting services as barriers to implementation).[46]

### Strengths and limitations

The case study design facilitated careful comparative analysis across national settings. PLA was an effective methodology for engaging migrants and other stakeholders from primary care in implementation research. However, while the sample had representation from the desired range of stakeholder groups it did not include migrants who currently experience language problems, which is a clear limitation. Involving clinicians and primary care practice staff in research was challenging, as is often the case [47]). The time consuming nature of PLA, as well as the engagement of different stakeholders in the PLA dialogue was sometimes challenging, as reflected in the fact that GPs in Greece and The Netherlands did not want to include migrants in meetings about practice organisation.

We had a rich data set comprised of fieldwork transcripts and researchers’ reports and we which enhanced our ability to understand the specifics of each national setting. We prospectively applied the theoretical framework NPT for structuring our analysis, and employed standard techniques enhancing the quality and rigour of the analysis.

The generalizability of our findings could be considered limited, given their qualitative nature. However, the aforementioned strengths of our comparative case study and the range of participants across settings has identified multiple transferrable points.

### Implications for research and practice

We recommend involving migrants and other key stakeholders when adapting, introducing and evaluating interventions designed to improve cross-cultural communication in primary care settings. The involvement of all clinical and administrative practice members in G/TIs training on cultural competence, and consideration of the evaluation of training, appears to enhance buy-in to future practice changes.

Further research is needed into the effects of these interventions on practitioner knowledge and behaviour as well as migrants’ health outcomes. We also need to know more about the best methods of including migrants who currently experience language problems within their host country [48, 49] and into the quality and appropriate form of interpretation services [50, 51]. Finally, more intersectoral collaborative work is needed to identify solutions to problems relating to policy, funding and other related macro level factors.

## Conclusions

Successful implementation of G/TIs to improve communication in cross-cultural general practice consultations benefits from mutual engagement between migrants and other key stakeholders [52], flexible adaptation of G/TIs to meet local needs before their introduction; and collaborative evaluation after a period of time in use.

## Abbreviations

G/TIs: Guidelines and training initiatives
GP: General practitioner
NPT: Normalisation process theory
PLA: Participatory learning and action
RESTORE: REsearch into implementation STrategies to support patients of different ORigins and language background in a variety of European primary care settings
